# Mechanisms and management considerations of parent-chosen feeding approaches to infants with swallowing difficulties: an observational study

**DOI:** 10.1038/s41598-021-99070-w

**Published:** 2021-10-07

**Authors:** Sudarshan R. Jadcherla, Kathryn A. Hasenstab, Erika K. Osborn, Deborah S. Levy, Haluk Ipek, Roseanna Helmick, Zakia Sultana, Nicole Logue, Vedat O. Yildiz, Hailey Blosser, Summit H. Shah, Lai Wei

**Affiliations:** 1 The Innovative Infant Feeding Disorders Research Program, Center for Perinatal Research, The Research Institute at Nationwide Children’s Hospital, Columbus, OH USA; 2 Division of Neonatology, Nationwide Children’s Hospital, 575 Children’s Crossroads, Columbus, OH 43215 USA; 3 Department of Pediatrics, The Ohio State University Wexner Medical Center, Columbus, OH USA; 4 Department of Speech and Language Pathology, Hospital de Clínicas de Porto Alegre, Department of Health and Communication, Universidade Federal do Rio Grande do Sul, Porto Alegre, Brazil; 5 Biostatistics Resource at Nationwide Children’s Hospital (BRANCH), Nationwide Children’s Hospital, Columbus, OH USA; 6 Center for Biostatistics, Department of Biomedical Informatics, The Ohio State University College of Medicine, Columbus, OH USA; 7 Division of Clinical Therapies, Nationwide Children’s Hospital, Columbus, OH USA; 8 Department of Radiology, Nationwide Children’s Hospital, Columbus, OH USA

**Keywords:** Paediatric research, Gastroenterology

## Abstract

Videofluoroscopy swallow studies (VFSS) and high-resolution manometry (HRM) methods complement to ascertain mechanisms of infant feeding difficulties. We hypothesized that: (a) an integrated approach (study: parent-preferred feeding therapy based on VFSS and HRM) is superior to the standard-of-care (control: provider-prescribed feeding therapy based on VFSS), and (b) motility characteristics are distinct in infants with penetration or aspiration defined as penetration-aspiration scale (PAS) score ≥ 2. Feeding therapies were nipple flow, fluid thickness, or no modification. Clinical outcomes were oral-feeding success (primary), length of hospital stay and growth velocity. Basal and adaptive HRM motility characteristics were analyzed for study infants. Oral feeding success was 85% [76–94%] in study (N = 60) vs. 63% [50–77%] in control (N = 49), *p* = 0.008. Hospital-stay and growth velocity did not differ between approaches or PAS ≥ 2 (all *P* > 0.05). In study infants with PAS ≥ 2, motility metrics differed for increased deglutition apnea during interphase (*p* = 0.02), symptoms with pharyngeal stimulation (*p* = 0.02) and decreased distal esophageal contractility (*p* = 0.004) with barium. In conclusion, an integrated approach with parent-preferred therapy based on mechanistic understanding of VFSS and HRM metrics improves oral feeding outcomes despite the evidence of penetration or aspiration. Implementation of new knowledge of physiology of swallowing and airway protection may be contributory to our findings.

## Introduction

Infants with feeding difficulties can be described as having frequent aerodigestive symptoms such as coughing, apnea, bradycardia, or desaturation with oral feeding, or the inability to achieve exclusive oral feeding. Overall, prevalence of infant feeding difficulties is increased and rising in medically complex infants^1,2^. Establishment of safe feeding is required prior to discharge^3,4^. In infants with feeding difficulties or frequent symptoms, it is common practice to assess the infants’ eating skills via dynamic x-ray imaging or videofluoroscopy swallowing study (VFSS) to provide structural and functional insight^5,6^. If penetration or aspiration is observed during the VFSS, feeding modifications are trialed and typically include nipple flow rate or fluid thickness changes further increasing the infant’s radiation exposure and associated risks^7–9^. Additionally, standardization is still being developed for infants^10,11^, and evaluation is typically limited to observation of the oral cavity and upper aerodigestive tract as well as only those swallows as captured by the radiologist. High resolution manometry (HRM) is an emerging technology in infants that permits prolonged evaluation of swallowing function without radiation exposure^12–20^. HRM allows evaluation of swallowing function by examining dynamic and kinetic relationships between the airway (glottal closure and respiratory changes) and the entire foregut (pharynx, upper esophageal sphincter- UES, esophagus, lower esophageal sphincter- LES).

As HRM may be complementary to VFSS, the aim of the current study was to test the main hypothesis that clinical outcomes of an integrated feeding approach (parental preference informed by VFSS and HRM testing) are superior to the standard-of-care approach (control) based on VFSS information alone. A sub-aim was to test the hypothesis that infants with penetration or aspiration have distinct clinical and motility outcomes.

## Participants, study design and methods

### Study design, setting, participants

This is an observational cohort study conducted between 2015 and 2020 at a single tertiary all-referral center at the Nationwide Children’s Hospital, Columbus, OH, in infants referred for feeding difficulties and VFSS evaluation. In accordance to institutional guidelines and regulations involving human subjects, the protocol was approved by the Institutional Review Board at Nationwide Children’s Hospital, Columbus, OH (Supplement). The study was registered on clinical trials.gov: NCT02583360. Originally, this study was designed as a randomized clinical trial to compare the effects of thickened formula vs nipple flow change feeding modifications in infants undergoing VFSS evaluation. Initially, recruitment was difficult owing to the institution of family-centered care policy, compounded by provider variability with feeding therapeutic strategies, and lack of provider and parental support for randomization. Therefore, based on the advice of the data safety monitoring board and study sponsor, alternative strategies were employed (parent preferred feeding therapies and inclusion of outpatient populations), and study design was modified to the current observational cohort design (study: prospectively collected integrated feeding approach vs control: retrospectively collected standard-of-care approach) to address the original study goals of identifying potential mechanisms and management strategies for infant dysphagia. The integrated feeding (study) approach included parent chosen feeding therapies based on information provided from VFSS and HRM assessments, while the standard-of-care (control) approach included provider-driven therapies based on VFSS only. Feeding therapies for both groups included nipple flow, fluid thickness, or no modification. For the study cohort: Subjects were screened and recruited by the Neonatal and Infant Feeding Disorders Research Program. Written, signed, informed parental consent was obtained prior to the study. Parents were encouraged to attend HRM evaluations, participate in feeding sessions with nipple and fluid thickness changes, and ask questions. Regardless of attendance, parents were educated about their infant’s swallowing limitations and capabilities via explanation of pharyngo-esophageal motility, airway protection, volume intake, and vital sign observations. For the control group: parental consent was not needed (as determined by the Institutional Review Board) as this was a medical record chart review of data from infants receiving the standard-of-care during the concurrent study time period. *Inclusion criteria for study and controls* were: (a) infants with feeding-related aerodigestive symptoms undergoing a diagnostic VFSS, (b) < 60 weeks postmenstrual age (both pre-term and full-term born), (c) on full enteral feeds with at least partial oral feeds, and (d) on ≤ 1 L per minute oxygen via nasal cannula for respiratory support. *Exclusion criteria for study and controls* were known genetic, metabolic or syndromic diagnoses: severe neuropathology (≥ grade III intraventricular hemorrhage, neurosurgery, moderate to severe perinatal hypoxic ischemic encephalopathy), gastrointestinal malformations and/or surgery, craniofacial malformations or ear/nose/throat surgeries, and exclusively breastfeeding infants.

### Videofluoroscopy swallow study protocol

Using the established evaluation procedures^21^ and institutional interdisciplinary guidelines of quality and safety^11^, VFSS was performed with 3 standardized metrics: field of view, magnification, and pulse repetition rate. Briefly, standardized collimation (lips anteriorly, inferior bony orbits superiorly, spinous processes posteriorly, cervical 5 and 6 vertebrae inferiorly) during a VFSS provided a focused view of the oropharyngeal tract and at least one bolus into the esophagus following it through while avoiding unnecessary radiation exposure to radiosensitive organs^22^. Magnification was ensured to provide anatomic detail but was balanced with the As Low as Reasonably Achievable principle to avoid unnecessary radiation exposure^11^. A magnification of not exceeding twice magnifications on a standard-three scale was used, but the majority of studies performed without any magnification^22^. Imaging was carried out in real time of 30 frames per second^23^. Infant was studied in their typical feeding position, which included side-lying or semi-reclined via a seat (Tumble Forms Feeder Seat Positioner, Patterson Medical, Illinois, USA). Imaging was performed by the radiologist in lateral view during bottle feeding. The infant was bottle fed premixed liquid barium sulfate (Varibar®, Bracco Diagnostics Inc, New Jersey, USA) by the caregiver or occupational therapist. Nipple flow rates and testing liquid thickness (thin, nectar, and thin honey) were determined based on the patient’s clinical needs. As per institutional standard of care, the VFSS team (an occupational therapist, speech language pathologist, and radiologist) performed assessment of oral, pharyngeal, laryngeal, and esophageal regions in real-time which were also verified through post-review for agreement.

### Pharyngo-esophageal motility testing protocol

Infants underwent motility testing via HRM as previously published^19,24,25^. Briefly, a 6 Fr probe with 25 pressure sensors (UniTip High-Resolution Catheter, Unisensor USA) attached to a portable HRM System (Solar GI, Laborie Medical Technologies, Mississauga, ON, Canada and Duracell PowerSource 1800, Duracell Incorporated, Connecticut, US) was zeroed prior to placement, passed nasally, and secured by the study physician at the patient’s bedside. The infant was given adequate time for catheter adaptation (≥ 15 min) to ensure quiescence before recording basal manometry and spontaneous swallows. Nasal airflow thermistor (Integra Life Sciences, Plainsboro, NJ) was utilized to detect respiratory changes and deglutition apnea^26–29^. VFSS testing as described above was performed concurrently when feasible (N = 54 infants) or sequentially within 7 days (N = 6 infants). If concurrent VFSS and HRM studies occurred, the infant was transported by the study team (physician, registered nurse, two technicians) to the VFSS suite for testing with vitals constantly monitored. Upon VFSS completion, the infant was transported back to the patient room where pharyngeal infusion protocol and oral feeding challenge were performed^19,30,31^. To perform pharyngeal infusions, a silicone catheter with pharyngeal infusion port (Dentsleeve International, Mui Scientific, Mississauga, ON, Canada) was juxtaposed to the HRM catheter and positioned so that the pharyngeal infusion port was at the level of the pharynx as confirmed by esophago-pressure topography plots in HRM. Sterile water was infused at volumes of 0.1, 0.3 and 0.5 mL in triplicate to evaluate pharyngeal swallowing reflexes. The oral feeding challenge consisted of a 1-min milk bottle feed using the infant’s current bottle and nipple system along with their current formula or human milk. Trial start time begun upon infant latch.

### VFSS and HRM data analytical methods

Analytical definitions for VFSS and HRM methods have been previously published^18,24,25,29,32–49^, are summarized in Table 1, and further explained below.

**Table 1:** Videofluoroscopy swallow study and high resolution motility metrics and analysis definitions.

Anatomic region	Variable name	Unit of measure	Measure type	Testing state				Definition
				*BS*	*Px*	*Milk feed*	*Barium feed*	
**Videofluoroscopy swallow study (based on the infant’s thinnest trial with worst PAS score)**								
Oral Cavity	Oral phase	%	Categorical				✓	*functional:* adequate lip closure, sucking strength, bolus formation, and transit time prior to initiation of pharyngeal swallow *delayed but functional:* delayed lip seal, bolus formation, transit time *impaired:* absent/reduced lip seal, sucking strength, bolus formation, transit time^43^
Pharynx	Pharyngeal phase	%	Categorical				✓	*functional:* transport of the bolus through the pharynx initiated by hyo-laryngeal elevation *delayed but functional:* entry of the bolus head into the pharyngeal cavity prior to hyo-laryngeal elevation/decreased laryngeal vestibule closure resulting in inconsistent shallow penetration *impaired:* reduced hyo-laryngeal elevation, incomplete closure of the laryngeal vestibule, reduced glottal closure resulting in consistent deep laryngeal penetration and/or aspiration before, during or after the swallow^43–45^
Larynx	Laryngeal phase	#	Categorical				✓	*Penetration-Aspiration Scale (PAS)* 1- material does not enter the airway 2- material enters the larynx but stays above the vocal folds 3- material enters the larynx to the level of the vocal folds 4- material passes below the vocal folds 5- material enters the airway, contacts the vocal folds, and is not ejected from the airway, 6- material enters the airway, passes below the vocal folds and is ejected into the larynx or out of the airway 7- material enters the airway, passes below the vocal folds, and is not ejected from the trachea despite effort, and 8- material enters the airway, passes below the vocal folds, and no effort is made to eject^49^
Larynx	Laryngeal phase	%	Categorical				✓	*PAS Group* *No Penetration or Aspiration:* PAS = 1 *Penetration:* PAS = 2–5 *Aspiration:* PAS = 6–8^49^
**High resolution manometry**								
Pharynx to Stomach	Peristaltic response occurrence	%	Categorical		✓			Presence of pharyngeal reflexive swallow or pharyngo-UES-contractile reflex^38,40–42^
Pharynx to Stomach	Peristaltic response latency	sec	Continuous		✓			Time interval between pharyngeal infusion onset and peristaltic response onset^25,39^
Pharynx to Stomach	Peristaltic response duration	sec	Continuous		✓			Time interval between peristaltic response onset and offset^25,39^
Pharynx to Stomach	Terminal swallow occurrence	%	Continuous		✓			Presence of final clearing pharyngo-esophageal swallow resulting in aerodigestive quiescence^25,39^
Pharynx	Pharyngeal contractions	#	Continuous		✓			Total number of pharyngeal contractions induced by pharyngeal infusion stimulus^29,38,39^
Pharynx	Pharyngeal contractile activity	%	Continuous			✓	✓	Sum of pharyngeal contractile durations/oral feeding duration*100^18,34^
Oro-Pharynx	Pharyngeal contractile vigor	mmHg.cm.s	Continuous	✓		✓	✓	Contractile integral calculated as pharyngeal region amplitude*pharyngeal length*contractile duration for proximal, distal, and overall pharyngeal regions. Proximal contractile integral reflects oro-pharyngeal functional competency^18,34,46^.
UES and LES	Basal tone	mmHg.cm.s	Continuous	✓				Contractile integral (amplitude*length* duration) calculated during a 2 s window at rest prior to basal swallow^36,37^
UES and LES	Relaxation reflex occurrence	%	Categorical		✓			Relaxation defined as > 50% decrease from basal tone^34,35^
UES	Contractile reflex	%	Categorical		✓			Contraction defined as > 50% increase from basal tone- definition adapted from previous works ^32,33^
Esophagus	Esophageal contractile vigor	mmHg.cm.s	Continuous	✓		✓	✓	Contractile integral (amplitude*length* duration) of esophageal regions. Proximal esophagus: lower UES border to transition zone. Distal esophagus: transition zone to upper LES border^24,25^
Esophagus	Peristaltic break during terminal swallow occurrence	%	Categorical		✓			Presence of any esophageal gaps in the 20-mmHg isobar contour of the peristaltic contraction associated with the terminal swallow^24,48^
Nasal airflow	DA occurrence	%	Categorical		✓			Presence of a pause in breathing associated with pharyngeal contraction^38,40^
Nasal airflow	DA latency	sec	Continuous		✓			Time interval between pharyngeal stimulus onset to DA onset^38,40^
Nasal airflow	DA duration	sec	Continuous	✓	✓			Time interval between respiratory pause onset to offset^38,40^
Nasal airflow	DA during interphase occurrence	%	Categorical		✓			Phase of deglutition apnea onset: Inspiration- upstroke in nasal airflow. Expiration- defined as downstroke in nasal airflow thermistor. Interphase- between inspiratory or expiratory phases^40^
Global	Symptom occurrence	%	Categorical		✓			Defined as the presence of any symptom during pharyngeal infusion ^47^

BS-Basal Swallow, Px-Pharyngeal Infusion, DA- deglutition apnea, Milk Feed- Oral Feeding with Milk, Barium Feed- Oral Feeding with Barium-Sulfate, ✓: variable was analyzed for marked state.

VFSS metrics were based on the infant’s thinnest trial received with the worst PAS score. The oral phase of swallow refers to structural and functional observations of the oral preparatory and oral transit stages of swallow prior to initiation of the pharyngeal phase^43^, the pharyngeal phase of swallow refers to observations of swallowing as the bolus enters the pharyngeal cavity, bypassing the closed laryngeal region, and exiting the pharyngeal cavity at the level of the UES^43,44,50^, and laryngeal phase and airway protection was assessed by the Penetration Aspiration Scale (PAS)^49^. Infants were also categorized as PAS = 1 (no penetration or aspiration) or PAS ≥ 2 (penetration or aspiration).

#### HRM metrics

HRM metrics were analyzed during resting and adaptive states as follows: (A) Basal Swallowing (resting state): For each study a maximum of ten basal or spontaneous (absence of stimulus) swallows were selected for analysis^37,51^. Pharyngeal, esophageal, and respiratory characteristics were analyzed as previously published^18,19,24,25,38^. (B) Pharyngeal infusion (adaptive state): aerodigestive responses to pharyngeal stimulus were analyzed as previously published and included global (peristalsis and symptoms) and regional (pharynx, UES, esophagus, respiratory) characteristics^24,25,29,38,39,41,42,47,52^. (C) Oral feeding with milk and barium sulfate (adaptive state): Pharyngeal and esophageal characteristics were measured during oral feeding sessions^24,25^. Volume intake was calculated as volume consumed (mL)/feeding session duration (min).

## Statistical methods and outcome measures

**Figure 1:**
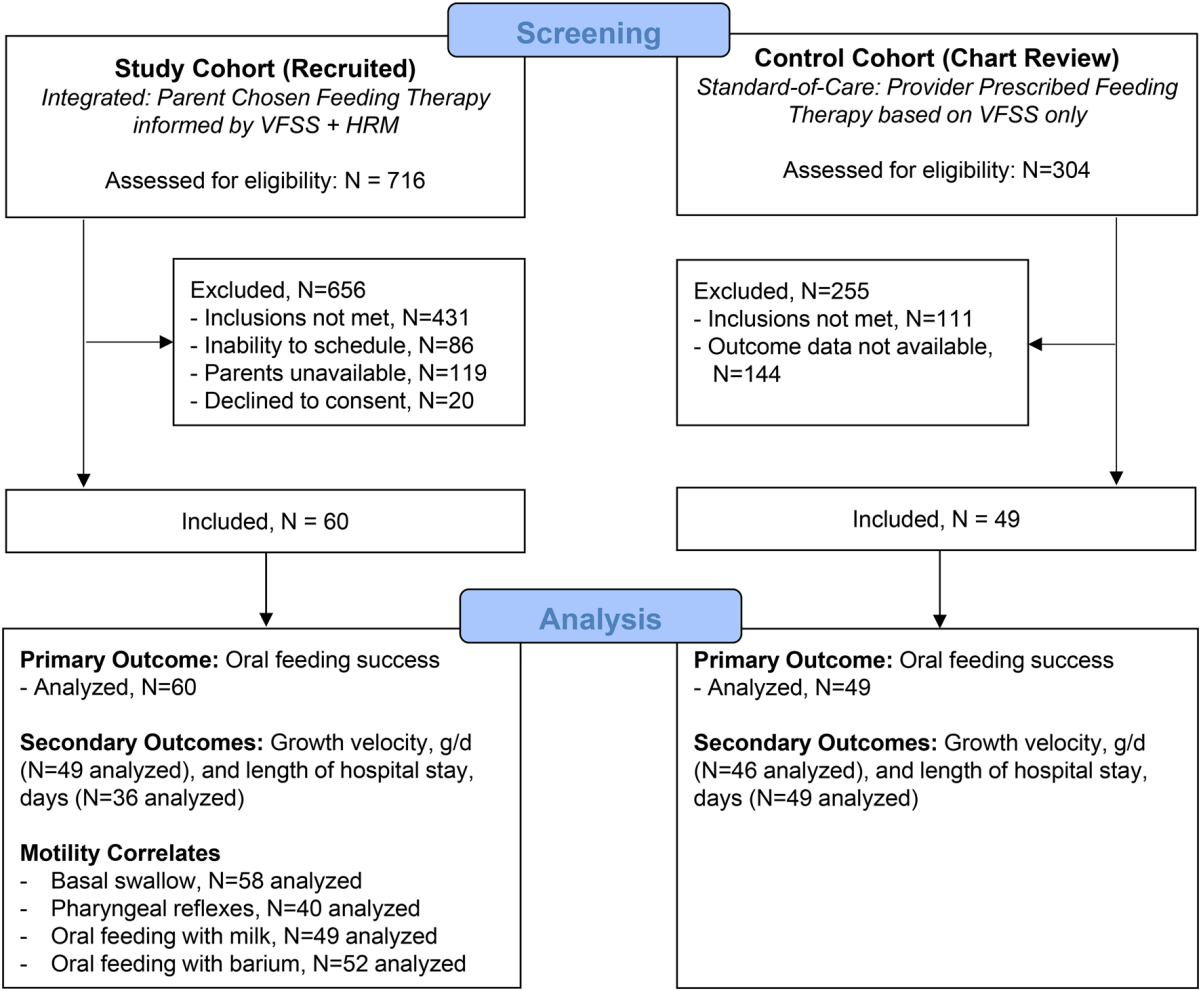
Study Enrollment. Depicted is the study flow diagram for analysis of study (prospectively collected) and control (retrospectively collected) cohort data for infants referred for VFSS testing.

Demographic and clinical outcomes were managed using research electronic data capture tools (REDCap)^53^. The primary clinical outcome was oral feeding success (defined as full oral feeding without aerodigestive feeding-related symptoms) at discharge or 4 weeks (whichever was sooner) for inpatients and 4 weeks for outpatients for study and control cohorts. Sample size of the study group was determined apriori as follows: based on historical data where oral feeding success rate was 60% with VFSS dependent treatment. With 60 patients enrolled, we had at least 90% power to detect an absolute increase of 20% from 60% for those who did not have treatment informed by manometry (historical control) to 80% in those in those informed by manometry with two-sided type I error of 0.05. Secondary clinical outcomes were growth velocities (weight, length, and head circumference) and length of hospital stay. Analysis of secondary outcomes was based on available data with number of subjects as stated in the tables and flow diagram (Fig. 1).

Statistical Analysis Software (v. 9.4, SAS Institute Inc., Cary, NC, USA) was utilized for analysis. *P*-values of < 0.05 were considered statistically significant. Descriptive statistics were reported as median [IQR], mean ± SD or total number and percentage for demographics and clinical characteristics. Oral feeding success rate was estimated with 95% Confidence Interval and compared between cohorts using Chi-squared test. Odds ratio with 95% confidence interval was also provided for oral feeding success. Logistic regression was used to calculate the adjusted odds ratio for oral feeding success while controlling for gastroesophageal reflux disease and prematurity to account for potential confounders, as these subject morbidities have been shown to delay feeding milestones. Two-sample t-test or Wilcoxon signed rank test for the continuous variables and chi-squared or Fischer’s exact tests for the categorical variables, whichever was appropriate, were used to compare the clinical and VFSS characteristics between study and control cohorts, and clinical outcomes between PAS = 1 and PAS ≥ 2 groups within and between study and control cohorts. Normality was assessed using Shapiro-Wilks test and visually inspection of the Q-Q plot (normality) and residual plots. For comparison of HRM motility characteristics between PAS = 1 and PAS ≥ 2 groups, linear mixed effect model for continuous measured variables and generalized estimating equation for categorical variables, to predict the likelihood of the specific response, were used. Both models controlled for presence of gastroesophageal reflux disease and prematurity. Pharyngeal infusion data was also controlled for infusion volume. Compound symmetry was specified for the covariance structure of the repeated data. Bonferroni correction was used for multiplicity adjustment to conserve the overall type I error at α = 0.05.

### Study oversight

Compliance to protocol and data integrity were maintained. Patient care data was stored and secured. Study recruitment criteria were reported to the data safety monitoring board quarterly (see composition in acknowledgment) and their recommendations complied. Clinical study progress and adverse events were reported to the institutional review board annually. In addition, voluntary audits were conducted at the request of the principal investigator by the institutional audit team (see acknowledgement) and recommendations complied. Outcome variables were documented as route of intake oral or tube, growth metrics, administration of acid suppressive therapies, supplemental oxygen use, adverse events and the data were confirmed using electronic medical records (Epic, Epic Systems Corporation, Verona, WI, USA) as well as parental validation.

## Results

### Comparison of outcomes in study cohort vs. control cohort

Study enrollment, and approaches for primary outcome analysis are described in the study flow diagram (Fig. 1)*.* Subject characteristics at birth and time of evaluation did not significantly differ between study and control cohorts (Table 2). Additionally, for study vs control groups respectively: Birth APGAR score, median [IQR], at 1 min was 6^3 – 8^ vs 7^4 – 8^, *p* = 0.53 and at 5 min was 8^7 – 9^ vs 8.5^7 – 9^, *p* = 0.46. Feeding therapies (nipple flow modification: fluid thickness modification: no modification, %) were 21: 22: 57 for study vs 23: 33: 44 for control, *p* = 0.35. Parental attendance was 46/60 (77%) in the study group and 27/48 (56%) in the control group, *P* = 0.30.

**Table 2:** Clinical and VFSS characteristics between Study vs. Control Cohorts.

Characteristic	Study	Control	*P*-value
	(N = 60)	(N = 49)	
**Demographics at birth**			
Gender [male] (%)	34 (57%)	19 (39%)	0.06
Race (%)			0.1
African American	9 (15%)	15 (31%)	
Asian	1 (2%)	2 (4%)	
Bi-racial	0 (0%)	1 (2%)	
White	50 (83%)	31 (63%)	
Gestational age (wks)	34.8 ± 4.8	35.7 ± 4.2	0.29
Birth weight (kg)	2.5 ± 1.1, n = 58	2.7 ± 1.0, n = 47	0.51
**Clinical characteristics at evaluation**			
Chronologic age (wks)	10.9 ± 6.0	10.0 ± 6.5	0.41
Postmenstrual age (wks)	45.7 ± 5.5	45.7 ± 5.1	0.95
Weight (kg)	4.4 ± 1.1	4.1 ± 1.0	0.14
Infant feeding milk type (%)			0.88
Breast milk	7 (12%)	5 (10%)	
Breast milk + formula	17 (28%)	16 (33%)	
Formula	36 (60%)	28 (57%)	
Morbidity (%)			
Preterm birth	34 (57%)	23 (47%)	0.31
Chronic lung disease of infancy	14 (23%)	8 (16%)	0.36
Intraventricular hemorrhage (grade I or II)	5 (8%)	4 (8%)	0.97
Hypoxic ischemic encephalopathy (mild)	1 (2%)	0 (0%)	0.36
Gastroesophageal reflux disease	20 (33%)	25 (51%)	0.06
**VFSS characteristics at evaluation**			
Feeding position [semi-reclined] (%)	44/57 (77%)	36/48 (75%)	0.79
Oral phase (%)			0.98
Functional	29/57 (51%)	24/48 (50%)	
Delayed but functional	25/57 (44%)	21/48 (44%)	
Impaired	3/57 (5%)	3/48 (6%)	
Pharyngeal phase (%)			0.64
Functional	32/57 (56%)	23 (47%)	
Delayed but functional	17/57 (30%)	18 (37%)	
Impaired	8/57 (14%)	8 (16%)	
Penetration aspiration scale (PAS) #	2 [1–7], n = 58	2 [2–8]	0.6
PAS category (%)			0.93
No penetration/aspiration (PAS: 1)	15/58 (26%)	12 (24%)	
Penetration (PAS: 2–5)	26/58 (45%)	21 (43%)	
Aspiration (PAS: 6–8)	17/58 (29%)	16 (33%)	

Data presented as n (%), mean ± SD, or median [IQR]. Chronic lung disease of infancy was defined as oxygen use at 36 weeks for infants born ≤ 36 weeks gestational age and oxygen need at discharge for infants born > 36 weeks gestational age. Gastroesophageal reflux disease diagnosis was presumed if treated with acid suppression.

**Figure 2:**
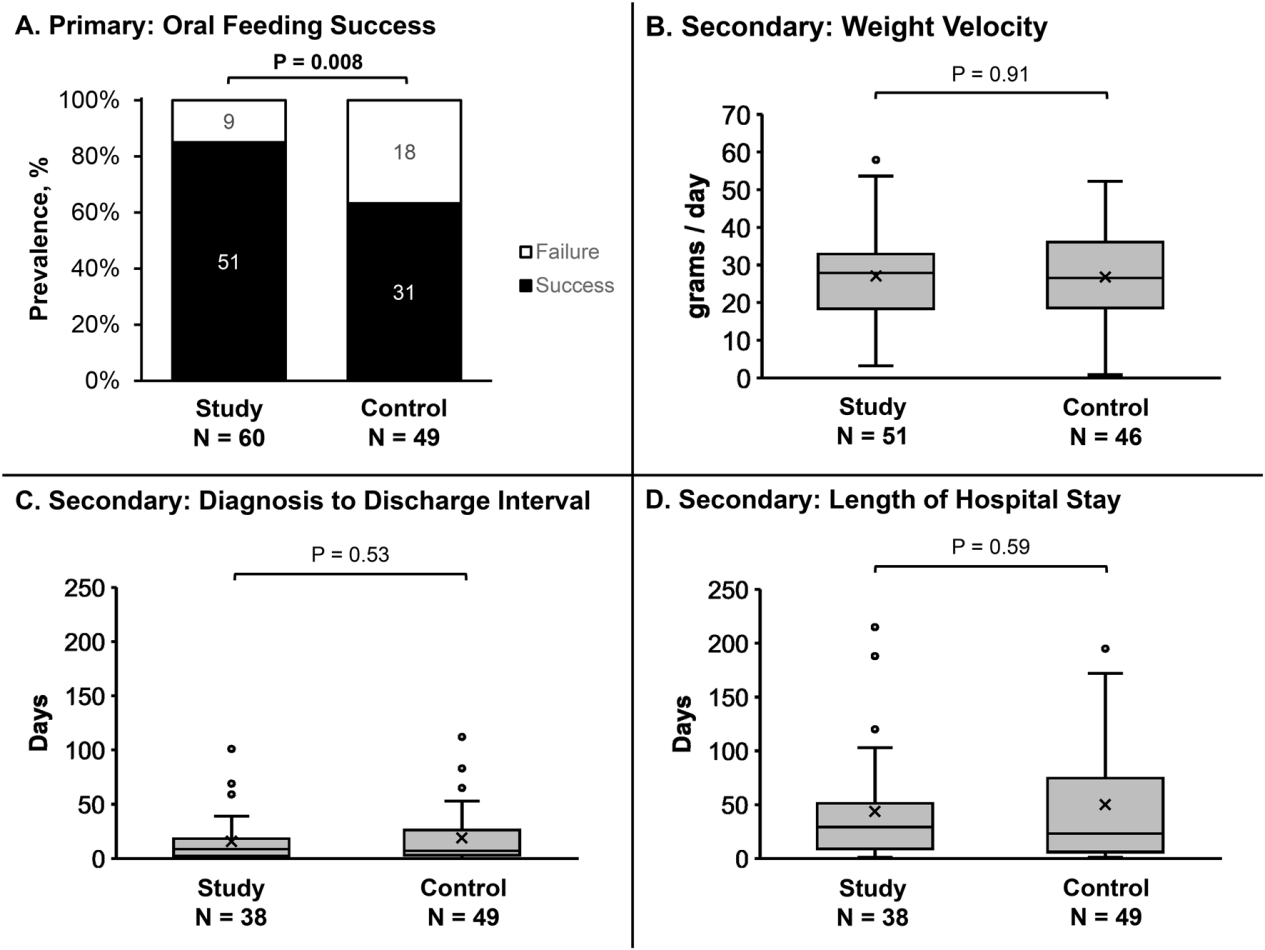
Clinical Outcomes of Infants referred for VFSS (Study Approach: VFSS + HRIM + Parent Preference) and Control (standard-of-care: VFSS informed). On the boxplots, X 's represents the mean while dots represent outliers. Primary clinical outcome success was greater in the study group (**A**). Secondary outcomes did not significantly differ (**B**–**D**). In figures (**C**,**D**), there were 22 infants in the study group studied as outpatients and discharged the same day, hence not included in the N value.

Primary and secondary clinical outcomes are shown in Fig. 2 with primary outcome 85% [76–94%] in study vs 63% [50–77%] in control [unadjusted OR: 3.29 (1.32–8.23), *p* = 0.008]. After adjusting for gastroesophageal reflux disease and preterm birth, study group was still more likely to achieve oral feeding success [adjusted odds ratio: 4.05 (1.49–10.95), *p* = 0.005]. Secondary clinical outcome growth velocities (cm/day) were 0.11 ± 0.07 vs 0.11 ± 0.06, *p* = 0.83 for length, and 0.07 ± 0.04 vs 0.06 ± 0.03, *p* = 0.17 for head circumference for study vs control, respectively.

### Effects of penetration or aspiration on clinical and HRM motility correlates

#### Clinical outcomes

**Figure 3:**
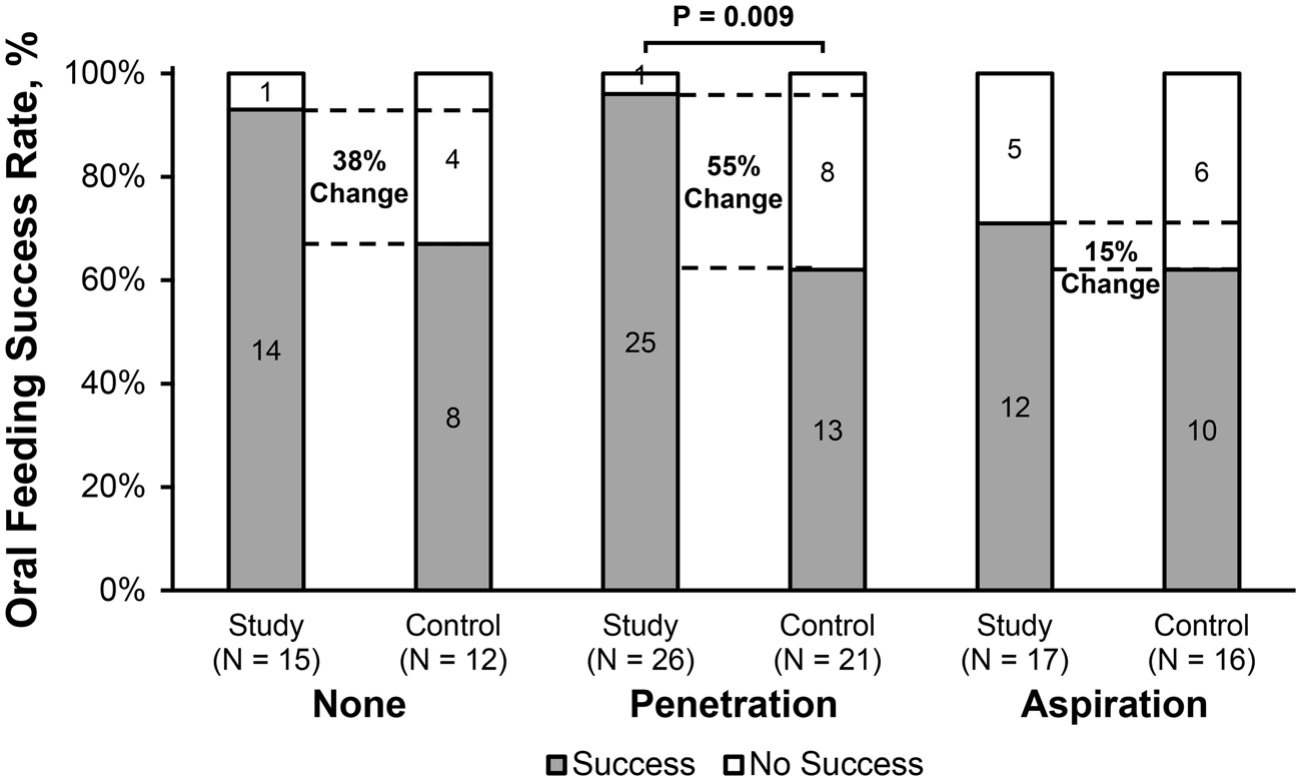
No penetration or aspiration: PAS = 1, Penetration: PAS = 2 to 5, Aspiration: PAS = 6 to 8. In the figure legend, success is defined as independent oral feeding, and no success as any tube feeding. Numbers within bars represent n-values of infants. Note in those infants with laryngeal penetration, feeding success was greater in the study group. Although not statistically significant, infants without penetration or aspiration may also clinically benefit from the study approach as indicated by 38% higher oral feeding success.

Primary and secondary clinical outcomes did not differ between (a) infants PAS ≥ 2 (vs PAS = 1) in both study and control cohorts, or (b) in study infants with PAS = 1 vs control infants with PAS = 1 (Table 3). However, in infants with PAS ≥ 2, oral feeding success was greater in the study group (Table 3), specifically driven by those infants with penetration (Fig. 3). Feeding therapies in infants with PAS = 1 did not differ between study and control groups (*P* = 0.30), as well as infants with PAS ≥ 2 between study and control groups (*P* = 0.29).

**Table 3:** Comparison of Clinical Outcomes within and between Study vs. Control Cohorts with and without Penetration or Aspiration.

Characteristic	Study			Control			PAS = 1: Study vs control *P*-value	PAS ≥ 2: Study vs control *P*-value
	PAS = 1 (None)	PAS ≥ 2 (Penetration or Aspiration)	Adjusted *P*-value	PAS = 1 (None)	PAS ≥ 2 (Penetration or Aspiration)	Adjusted *P*-value		
N-value	15	43		12	37			
Oral feeding success rate (%)	14 (93%)	37 (86%)	0.99	8 (67%)	23 (62%)	0.99	0.3	**0.04**
**Growth velocity**								
Weight (g/day)	27.6 ± 9.7, n = 9	27.3 ± 11.6, n = 40	0.99	27.3 ± 14.0, n = 11	26.6 ± 10.8, n = 35	0.99	0.99	0.99
Length (cm/day)	0.1 ± 0.1, n = 9	0.1 ± 0.1, n = 37	0.99	0.1 ± 0.0, n = 10	0.1 ± 0.1, n = 34	0.43	0.45	0.99
Head circumference (cm/day)	0.1 ± 0.0, n = 9	0.1 ± 0.0, n = 35	0.99	0.1 ± 0.0, n = 9	0.1 ± 0.0, n = 33	0.99	0.99	0.9
**Nutrition**								
Milk type (%)			0.83			0.99	0.34	0.99
Breast milk	2/15 (18%)	3/42 (7%)		1 (8%)	3 (8%)			
Breast milk + Formula	1/15 (9%)	12/42 (29%)		4 (33%)	8 (22%)			
Formula	8/15 (73%)	27/42 (64%)		7 (59%)	26 (70%)			
Oxygen at discharge* (%)	0/5 (0%)	11/31 (35%)	0.33	2 (17%)	5 (14%)	0.99	0.99	0.09
VFSS to discharge interval* (days)	3 [1–4], n = 5 (1–59)	9 [2–17], n = 31 (0–101)	0.99	9 [3–18.5] (0–114)	7 [3–27] (0–112)	0.99	0.99	0.99
Length of hospital stay* (days)	26 [20–38], n = 5 (2–123)	27 [9–63], n = 31 (1–215)	0.99	19 [7–99] (2–196)	26 [6–66] (1–198)	0.99	0.99	0.99

Data presented as n (%), mean ± SE, median [IQR], and (min, max). VFSS: videofluoroscopy swallow study, HRM: high resolution manometry. *Rates calculated for hospital inpatients only.

#### HRM motility

**Figure 4:**
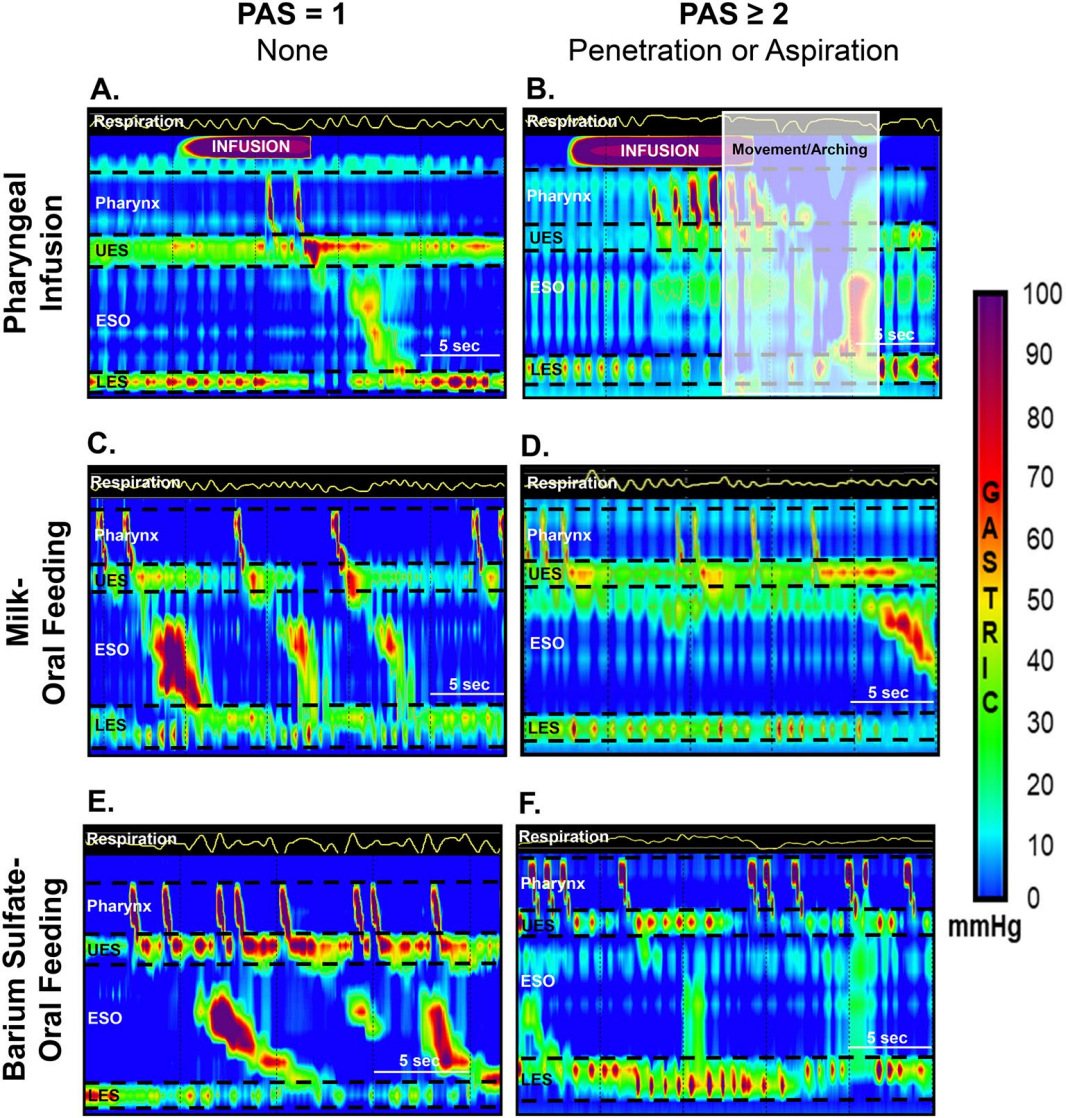
Motility correlates during pharyngeal infusion, oral feeding with milk, and oral feeding with barium sulfate of infants with and without penetration or aspiration. UES- upper esophageal sphincter, ESO- esophagus, LES- lower esophageal sphincter. Shown are representative esophago-pressure topography plots during HRM. Significantly, note in infants with penetration or aspiration symptoms are increased during pharyngeal infusion (**A,B**) and esophageal contractions are weaker during oral feeding with barium sulfate (**E,F**). Also note, stronger pharyngeal vigor during barium-sulfate oral feeding (**E,F**) vs milk oral feeding (**C,D**).

In study infants with PAS ≥ 2 (vs PAS = 1): (a) PAS score was 4^2 – 8^ vs 1^1 – 1^, *p* < 0.0001; (b) motility outcomes did not significantly differ for basal swallow or oral milk feeding (Table 4); (c) DA during interphase and symptoms were increased with pharyngeal infusions (Table 4); and (d) during oral feeding with barium sulfate, VFSS feeding duration was 85.6 ± 10.3 vs 124.5 ± 16.2 s, *p* = 0.048, and distal esophageal contractile vigor was decreased (Table 4). Also note during oral feeding, pharyngeal contractile vigor was greater with barium sulfate (vs milk) (Table 4). A representative HRM figure comparing infants with and without penetration-aspiration during pharyngeal infusion and oral feeding is shown (Fig. 4).

**Table 4:** Comparison of HRM motility characteristics in study infants with and without penetration or aspiration.

Characteristic	PAS = 1	PAS ≥ 2	*P*-value
	None	Penetration or Aspiration	
**Basal Swallow**	N = 15	N = 43	
Pharyngeal vigor (mmHg.cm.s)	121 ± 12	98 ± 8	0.11
Proximal vigor (mmHg.cm.s)	74 ± 10	55 ± 6	0.12
Distal vigor (mmHg.cm.s)	47 ± 7	42 ± 4	0.57
UES: basal tone (mmHg.cm.s)	28 ± 6	20 ± 3	0.2
Esophagus			
Proximal vigor (mmHg.cm.s)	80 ± 15	65 ± 9	0.41
Distal vigor (mmHg.cm.s)	337 ± 48	360 ± 28	0.68
LES: basal tone (mmHg.cm.s)	68 ± 10	62 ± 6	0.63
Respiratory: DA duration (s)	0.8 ± 0.1	1.1 ± 0.1	0.08
**Pharyngeal Reflexes**	N = 6	N = 34	
Peristaltic response occurrence	0.9 [95% CI 0.5–1.7]		0.7
Peristaltic response latency (s)	4.3 ± 0.7	4.8 ± 0.3	0.52
Peristaltic response duration (s)	16.7 ± 2.6	19.0 ± 1.1	0.42
Pharynx: total contractions (#)	4 ± 1	4 ± 0	0.47
UES			
Relaxation reflex occurrence	1.1 [95% CI 0.4–2.5]		0.89
Contraction reflex occurrence	0.8 [95% CI 0.3–2.2]		0.71
LES			
Relaxation reflex occurrence	2.4 [95% CI 1.0–5.9]		0.05
Respiratory			
DA occurrence	0.9 [95% CI 0.4–2.0]		0.71
DA latency, (s)	4.9 ± 0.8	4.9 ± 0.3	0.97
DA duration, (s)	1.1 ± 0.7	2.4 ± 0.3	0.1
DA during interphase occurrence	1.9 [95% CI 1.1–3.4]		**0.02**
Terminal swallow occurrence	0.7 [95% CI 0.4–1.2]		0.15
Esophagus: peristaltic break occurrence	3.4 [95% CI 0.9–13.6]		0.08
Symptom occurrence	2.5 [95% CI 1.2–5.3]		**0.02**
**Oral Feeding with Milk**	N = 14	N = 35	
Volume intake rate (mL/min)	6.1 ± 0.8	4.9 ± 0.6	0.25
Oral feeding duration (s)	70.6 ± 11.0	95.6 ± 7.0	0.06
Pharynx			
Contractile activity (%)	54.4 ± 14.9	52.0 ± 10.6	0.9
Vigor (mmHg.cm.s)	78 ± 10	76 ± 7	0.85
Proximal vigor (mmHg.cm.s)	44 ± 8	39 ± 5	0.65
Distal vigor (mmHg.cm.s)	33 ± 7	36 ± 5	0.69
Esophagus: Distal vigor (mmHg.cm.s)	432 ± 83	337 ± 49	0.33
**Oral Feeding with Barium Sulfate**	N = 15	N = 37	
Volume intake rate (mL/min)	10.9 ± 3.5	14.9 ± 1.9*	0.32
Oral feeding duration (s)	124.5 ± 16.2*	85.6 ± 10.3	**0.048**
Pharynx			
Contractile activity (%)	65.5 ± 7.2	60.4 ± 5.0	0.56
Vigor (mmHg.cm.s)	95 ± 10*	83 ± 8*	0.38
Proximal vigor (mmHg.cm.s)	51 ± 8*	42 ± 6*	0.35
Distal vigor (mmHg.cm.s)	44 ± 8*	42 ± 6*	0.82
Esophagus: Distal vigor (mmHg.cm.s)	460 ± 67	217 ± 45	**0.004**

Data presented as Mean ± SE or Odds Ratio [95% CI] with PAS = 1 used as reference group for Odds Ratios. Interpretation example: infants with penetration or aspiration are 2.5 times more likely to have symptoms than those without penetration or aspiration. UES- upper esophageal sphincter, LES- lower esophageal sphincter, DA- deglutition apnea.

*p < 0.05 versus oral feeding with milk.

## Discussion

### Overarching purpose, rationale, and goals

Delays with acquisition of safe oral feeding milestones often lead to non-objective “kitchen-sink” approaches, which result in increased length of hospitalization and therefore, health care costs. VFSS is widely available as a ‘gold standard’ radiological procedure to evaluate swallowing functions, but it can be highly subjective in the absence of standardization of testing process, analysis and recommendations. Prolonged provocative physiological testing like crib-side feeding methods while monitoring for symptoms is not possible with VFSS owing to the risks of radiation exposure. Presence or absence of aspiration or penetration during VFSS alone may not be adequate in developing feeding therapies. HRM permits prolonged provocative evaluation in the absence of radiation exposure under physiological conditions at crib-side while monitoring for pathophysiological changes. Furthermore, HRM is emerging as a safer method to assess not only swallowing pathophysiology but also feeding methods and aerodigestive protective reflexes regardless of underlying primary diagnosis. During HRM, oral feeding challenges can be permissible with various feeding systems including breastfeeding, when able. Some empiric approaches to manage feeding difficulties may include evaluation of aerodigestive apparatus for structural details, modifying nutrition or changing nipples, adding thickening agents, changing to breast milk substitutes, beginning gastric acid suppression, or adopting postural modifications^54^. Any of these methods may not improve outcomes, as feeding is a complex skill and involves understanding of the process, physiology, patient’s skills and airway protective mechanisms. Commonly, when there is failure with empiric approaches, discharge tube-feeding decisions (gastrostomy and or fundoplication, chronic nasogastric or transpyloric feeds) are made, and gastrostomy rates at discharge are increasing^55^. It is unknown how these diagnostic decisions and management strategies impact oral feeding success and hospital utilization in infants with and without penetration or aspiration, and how pharyngo-esophageal motility differs in infants with penetration or aspiration. Therefore, this study was undertaken to evaluate the effects of (a) an integrated study approach (VFSS and HRM guided decision making for therapy) vs the standard of care approaches (VFSS alone) on oral feeding outcomes and hospital utilization, and (b) penetration or aspiration on oral feeding outcomes, hospital utilization, and pharyngo-esophageal physiology.

### Salient findings of our study

Salient findings of the current study are as follows: (1) *In study vs control*: The study cohort was superior to the control cohort in achieving oral feeding success (primary outcome). Secondary clinical outcomes (growth velocities, nutrition, oxygen requirement at discharge, and length of hospitalization) did not significantly differ. Feeding therapies (nipple flow, fluid thickness, or no modification) did not significantly differ. (2) *In infants with penetration or aspiration (vs none)*: (a) clinical outcomes did not significantly differ, (b) sensory-motor motility characteristics (pharynx, UES, esophageal, and LES) did not significantly differ during basal swallowing or oral milk feeding, (c) DA during interphase and symptoms were more likely to occur during pharyngeal infusion, and d) distal esophageal contractile vigor was lesser during feeding with radiological contrast. (3) *Media effects:* Barium sulfate (vs milk) resulted in greater pharyngeal contractile vigor.

### Clinically important reasons for the study outcomes underlie in study approaches

Providing the additional mechanistic data with HRM at crib-side for a prolonged period, and having parents provide therapy based on their understanding of the combined results of VFSS and HRM findings may have improved the oral feeding outcomes in these complex infants. Empowering parents to make decisions for their infant’s feeding based on the expanded objective data likely led to better outcomes. On a different note, diagnostics can only improve treatments through the selection of several components of the therapeutic bundle, as eating is a complex process. We believe, our study findings address this.

Parent participation/attendance can be variable. However, parents see what is going on during the testing process and ask relevant questions related to feeding and cofounders that are impeding discharge planning. When they see the factual findings as they are happening with regards to swallowing, reflexes, airway protection, volume intake, and vital signs, they then see the capabilities and limitations of their infant. This approach may have improved their confidence to feed, and in some situations, parents could feed their infant during HRM. All these approaches improve parent competency with infant feeding.

Finally, several feeding positions are commonly attempted by mother (as in breast-feeding positions) or by parents and providers in bottle feeding positions. One important thing this study addresses is airway safety, regardless of the feeding position. Unfortunately, we are not powered enough to directly answer this question and additional mechanistic study designs are needed with larger patient numbers. There were no differences in positions between Study and Control (Table 2) infants at VFSS evaluation.

### Cross-systems physiology of glottal closure amidst esophageal clearance

In infants with penetration or aspiration, DA (a surrogate marker of glottal closure) was twice as likely to occur during interphase and was approximately twice as long (Table 4), which also translated to 2.5 times more symptoms. It is likely that in those infants with penetration or aspiration, the pause in breathing in between the respiratory phases is extended. With penetration, glottal closure is coordinated and effective in preventing bolus from entering below the vocal cords. With aspiration, coordination of glottal closure fails, where symptoms may occur, or may not occur and is termed “silent aspiration”. Glottal closure and pharyngo-esophageal clearance mechanisms have been described by us and others in infants with and without swallowing abnormalities^26–28,33,56,57^.

Central swallowing mechanisms are hierarchical, and are adaptational; for example, when these mechanisms are dysfunctional (as in the absence of swallowing, poor propagation, or poor coordination) other cascading reflexes are triggered such as coughing or apnea/bradycardia/desaturation events^26–28,33,57^. On the other hand, swallowing is also an important restorative mechanism for cardiorespiratory and aerodigestive homeostasis via effective terminal swallowing^28,29,33,38,39^. Thus, what construes as a troublesome symptom (problem) is actually a sign of adaptive skill in ensuring aerodigestive clearance. In the current study, terminal swallowing was present indicating that the capability exists in infants with penetration or aspiration. However, the presence of esophageal peristaltic breaks during pharyngeal infusion is trending towards significance and distal esophageal contractile vigor is significantly lesser in patients with penetration/aspiration, which are markers of esophageal dysmotility. Therefore, underlying issues maybe associated with dysfunctional esophageal motility and clearance mechanisms or peristaltic coordination, all of which are important components of pharyngo-esophageal propulsion, esophageal clearance and aerodigestive protection. Hence, potential therapeutic targets may be to strengthen esophageal motility mechanisms and cross-system interactions by prescribing oral feeding therapies cautiously. Additionally, as the current study evaluates swallowing function at the patient level (gross abnormalities), evaluation of individual swallows (acute abnormalities) resulting in penetration or aspiration may provide insight into sensory-motor physiologic vs pathophysiologic mechanisms of glottal closure and swallowing coordination.

### Implications for standardization of diagnostic and management approaches

*Suggested modifications to VFSS evaluation methods are as follows*: (a) Shortening Testing Duration: In infants with penetration or aspiration, VFSS trial duration was 85.6 ± 10.3 s indicating that if penetration or aspiration were to occur, it would likely be within this timeframe. In infants without penetration/aspiration, testing was prolonged by more than 30 s, thus increasing radiation exposure (Table 4: Oral Feeding with Barium Sulfate). Therefore, we suggest standardizing and limiting individual VFSS trials to less than 90 s because if aspiration were to happen, it would have in that time frame. (b) Consider changes to testing media: Oral feeding with barium sulfate may not be a physiologic comparator to milk feeding for evaluation of pharyngeal function as pharyngeal contractile vigor was greater with barium sulfate (vs milk) (Table 4). (c) Modified protocol to evaluate protective mechanisms in aspirators: True silent aspiration may result when symptoms do not occur and may be a marker of sensory dysfunction and gross failure of protective mechanisms. Studies have been controversial whether aspiration is truly detrimental. This may be due to operational testing conditions. Normally when the parent or feeding provider sees aerodigestive symptoms during feeding, nipple is immediately removed from the infant’s mouth, which likely triggers a terminal swallow and facilitates aerodigestive clearance, as has happened in our HRM study. Therefore, it is plausible that true silent aspiration is overestimated, and infant may not have met the sensory threshold to activate potential compensatory mechanisms.

*Addition of HRM to complement VFSS and improve mechanistic understanding and outcomes*: HRM testing includes prolonged and detailed evaluation of kinetic and dynamic swallowing-, breathing-, functional-, and aerodigestive protective mechanisms without the need for neonatal ICU patient transport or exposure to radiation. It also enables assessment of neurologic, cardio-respiratory, and swallowing rhythms in the presence or absence of symptoms. Thus, this approach can add value in improving the feeding and discharge outcomes without the risk of adverse events, even in those with laryngeal penetration and aspiration. Advanced research protocols and quality improvement initiatives can also emerge from this work in future in refining diagnostic and therapeutic strategies in the context of deglutition disorders and aerodigestive complications.

### Limitations/Future Directions

This study has limitations as follows: (1) Although randomized allocation of feeding modification therapies would have added scientific rigor (eliminating bias), it was not pragmatic owing to parent-provider hesitancy; hence, study design was modified. (2) The addition of HRM and the parent preferred therapy with concurrent controls provided important clinical outcome data, but control cohort did not have the benefit of HRM. (3) The current study evaluates gross swallowing function abnormalities at the patient level. Detailed evaluation of individual swallows resulting in penetration or aspiration is needed to detect acute swallowing abnormalities in real time. This would likely provide insight into sensory-motor physiologic vs pathophysiologic mechanisms of glottal closure and swallowing coordination. (4) While VFSS is frequently considered by physicians/therapists, there is a variability with the conduct of VFSS studies among neonatal ICU infants with regards to indications, timing, approach, analysis, and recommendations. (5) This study was conducted in a tertiary care referral center where we see complex feeding difficulties, and VFSS is frequently done for infant’s with dysphagia. However, given the superior outcomes using our innovative study approaches, the reliability of VFSS alone in developing long-term feeding strategies is questionable.

## Conclusions

The study cohort was superior to the control cohort in achieving oral feeding success in infants referred for VFSS evaluation. This indicates that comprehensive evaluation and individualized management strategies including parental education with feeding engagement practices may be more beneficial than prescribed feeding modifications based on VFSS alone. With the addition of HRM, establishment of compensatory mechanisms, modification of esophageal motility and airway interactions are potential therapeutic targets in infants with or without penetration or aspiration. Diagnostic and mechanistic evidence-based feeding management bundles can then be developed for the most appropriate and pragmatic care, thus resulting in superior clinical outcomes. These approaches may also provide confidence to parents with post-discharge feeding management among neonatal intensive care unit graduates.

## Data Availability

The datasets generated during and/or analyzed during the current study are not publicly available due to information that may compromise privacy of research participants, and further development of manuscripts are in process to address other project goals. These data may be available upon reasonable request to corresponding author.

## Abbreviations

VFSS: Videofluoroscopy swallowing study
HRM: High resolution manometry
PAS: Penetration aspiration scale
UES: Upper esophageal sphincter
LES: Lower esophageal sphincter
DA: Deglutition apnea
